# Recurrent thromboflebitis as a warning sign for cancer: a case report

**DOI:** 10.1186/1757-1626-2-153

**Published:** 2009-10-13

**Authors:** Henk CPM van Weert, Francien Pingen

**Affiliations:** 1 Academic Medical Centre Amsterdam, Dpt General Practice Meibergdreef 15, 1105 AZ Amsterdam, the Netherlands; 2 Health Centre Gein, Wisseloordplein 50 1106 MH Amsterdam, the Netherlands

## Abstract

**Introduction:**

The association between unprovoked deep venous thrombosis or pulmonary embolism and malignancy is well established. For unprovoked superficial thrombophlebitis this association has been documented in a few case-reports only.

**Case presentation:**

A 54-year-old apparently healthy male presents for the fourth time with an unprovoked superficial thrombophlebitis of the leg. When screened for underlying causes a renal cell carcinoma and an adenocarcinoma of the prostate are diagnosed. The renal cell carcinoma pointed out to have been visible on a CT-scan, made just before the time of the first presentation of the thrombophlebitis.

**Conclusion:**

Unprovoked and recurrent thrombophlebitis is a rare condition and its presentation might be a warning sign for a (yet undiagnosed) cancer. Physicians should be on their guard and consider screening for cancer. Usefulness of such a screening however is not known.

## Introduction

In 1865 Trousseau described the association between venous thromboembolism and malignancy for the first time. Originally he described an unexplained and migratory thrombophlebitis, but later the Trousseau's syndrome was used for the association of all kinds of hypercoagulable states and cancer [1]. Since then the association between venous thromboembolism (VTE: deep venous thrombosis and pulmonary embolism) and cancer has been confirmed in many prospective as well as retrospective studies. A patient with an unprovoked deep venous thrombosis or pulmonary embolism that is screened for cancer has a chance of 6% that a prevalent, previously undetected cancer is diagnosed and a 10% chance that previously undetected cancer is diagnosed within a year. The risk in unprovoked deep venous thrombosis or pulmonary embolism is about four times the risk of provoked disease [2]. The best screening strategy for undiagnosed cancer however is not known, as we do not know whether extensive screening has any impact on survival or quality of life compared with limited or no screening.

For superficial thrombophlebitis the association with cancer has been described in a few case reports only and has never been studied in well designed cohortstudies [3,4]. An exceptional form is Mondor's disease, in which phlebitis of the thoracic wall is associated with breast cancer [5].

## Case presentation

In the spring of 2001 a 51-year-old policeman consults his general practitioner (GP) with a painful, red spot on his upper limb. He is not ill, has no fever, has never smoked, uses a couple of beers each day and is known with hypertension, for which he is treated with atenolol ZOC 100 mg once daily. With a length of 1.86 meters and a bodyweight of 98 kilograms he is slightly over weighted.

The spot is there for a few days and looks like an oval shaped raised erythema. His GP diagnoses a superficial thrombophlebitis and prescribes conservative treatment: elevation of the leg and compresses. Within a few weeks the string disappears. Two years later the story repeats itself in exactly the same manner, but now the spot is located on the front of his left tibia. The GP again diagnoses superficial thrombophlebitis and prescribes a NSAID. The thromboflebitis again resolves itself in a few weeks.

A year later he consults again, this time with a swollen left calf, that started just above the ankle and a painful string at the inner side of the knee. Compression ultrasonography however shows no deep vein thrombosis, but again superficial thrombophlebitis. Treatment as before with NSAID. A year later he experiences a superficial thrombophlebitis for the fourth time (again diagnosed with compression ultrasonography) and a consulted specialist advocates screening on thrombophilia and possible malignancy. No thrombophilia is found, but PSA is slightly raised and because of a raised gamma-GT (123 U/l, which a month later spontaneously recovers to 63 U/l) a CT-scan of the abdomen is made. This reveals a solid mass in the left kidney and a solid mass in the suprarenal gland.

A partial nephrectomy and extirpation of the suprarenal gland is performed and biopsies of his prostate are taken. The pathologist diagnosis a renal cell carcinoma of the kidney, clear cell type with a diameter of 4.3 cm, Fuhrmann grade 111-IV, an adenoma of the adrenal cortex and adenocarcinoma of the prostate, Gleason score 6.

The patient recovers of the operation, distant metastases were not found. He is treated with prostatectomia for his prostate carcinoma. A year after the first operation a metastasis of the renal cell carcinoma is found in the right suprarenal gland, which is treated by extirpation. During the two years form his first to his third operation he suffered from deep venous thrombosis twice. Now, three years after the operation he has resumed working.

Retrospectively the renal mass was visible on a scan, made in 2001 because of his hypertension, that was difficult to treat at that time. This renal mass however was not noticed at that time.

## Discussion

As many as seven different types of vascular disorders, preceding the diagnosis of cancer have been described in the literature. Although the relation between phlebitis and cancer has been described before and can be explained in biological terms, only for deep vein thrombosis and pulmonary embolism this association is repeatedly confirmed in well designed epidemiological studies [6].

According to Virchow three factors are responsible for thrombosis: changes in the bloodflow, abnormalities of the blood vessel and changes in the composition of blood.

These three mechanisms might be responsible for the association between unprovoked VTE and cancer: invasion of cancer cells into the vessel wall, compression of the vasculature by a tumor, and hypercoagulability induced by the cancer. That the third mechanism is important is underscored by increased plasma levels of products of fibrin formation in cancer patients, like D-dimers. Biologically hypercoagulability as a paraneoplastic phenomenon is explained by circulating higher levels of substances as interleukin-2, produced by cancer-cells and increased autoantigens, originating from dying cancer cells [6].

As deep vein thrombosis superficial thrombophlebitis can be provoked (intravenous catheter = blood vessel or varicosis = bloodflow) and unprovoked. It seems reasonable that the same factors that cause VTE are responsible for the superficial variant of thrombosis: superficial thrombophlebitis. A status of hypercoagulability, as is found in cancer thus might well be responsible for both deep vein thrombosis as well as for superficial thrombophlebitis, that is not provoked by varicosis of intravenous catheterisation.

Our patient illustrates some important aspects of clarifying the relation between superficial thrombophlebitis and cancer: the development of cancer preceded the development of (recurrent) unprovoked superficial thromboflibitis, As we can never establish the causality of the relation between cancer and phlebitis in an experiment this temporal relation is important. Secondly the cancer was clinically not known at the time of presentation of the (multiple) episodes of superficial thrombophlebitis. Thirdly: as many erythemas are known to be related to cancer and superficial thrombophlebitis may resemble eryhthema a precise diagnosis is important [7]. Two of the four episodes of thrombophlebitis of our patient were documented objectively with ultra-sonography. And fourthly: all episodes of phlebitis were unprovoked.

## Conclusion

Our patient was a seemingly healthy person, apart from his hypertension. He was a policeman in active service and seldom absent from his work. The undiagnosed renal cell carcinoma was already present when he developed his first episode of superficial thrombophlebitis, as was clearly demonstrated with the revision of a scan, made because of his hypertension. For his prostate carcinoma we cannot be sure. Within five years he experienced four episodes of unprovoked superficial thrombophlebitis, that eventually led to the discovery of two different carcinomas.

Unprovoked superficial thrombophlebitis not only carries an increased risk for developing deep vein thrombosis[8] but it might also carry a risk for malignancy. Although empirical evidence for the association between superficial thrombophlebitis and malignancy still has to be established especially with recurrent disease doctors have to be on their guard and it might be worthwhile to screen patients with (recurrent) superficial unprovoked thrombophlebitis for malignancy. The best strategy however is not known and has to be weighed individually.

## Competing interests

The authors declare that they have no competing interests.

## Authors' contributions

FP analysed and described the medical history of the patient. HW did the literature research en wrote the final text. Both authors read and approved the final manuscript.

## Consent

Written informed consent was obtained from the patient for publication of this case report and accompanying images. A copy of the written consent is available for review by the Editor-in-Chief of this journal.
